# PREVALENCE OF INJURIES IN PROFESSIONAL FOOTVOLLEY ATHLETES

**DOI:** 10.1590/1413-785220253303e290454

**Published:** 2025-12-01

**Authors:** Maria Eduarda Dequi Diniz, Lucas Melo Neves

**Affiliations:** 1 Universidade de Santo Amaro, São Paulo, SP, Brazil.; 2 Universidade Estadual Paulista "Julio de Mesquita Filho" (UNESP), Instituto de Biociências, Departamento de Educação Física, Laboratório de Atividade Física, Esporte e Saúde Mental (LAFESAM), Rio Claro, São Paulo, SP, Brazil.

**Keywords:** Sports, Prevalence, Professional Athletes, Esportes, Prevalência, Atletas Profissionais

## Abstract

**Objective:**

Footvolley is a sport that have been gaining popularity, however, the literature on injuries in this modality is very scarce. Thus, this study aimed to describe the most common locations and types of injuries in professional footvolley athletes and compare the prevalence between male (M) and female (F).

**Methods:**

Observational, cross-sectional study, including the highest-ranked professional athletes in Brazil in the year 2023. The interviews took place during the national professional footvolley championship stages and included questions about sample characteristics, training history and routine, and injuries suffered throughout their career.

**Results:**

A total of 56 athletes, 36 F (26±5 years; 9±4 years of practice), and 20 F (28±6 years; 7±3 years of practice). Of this total, 95% reported some injury caused by playing footvolley, with knee injuries being the most prevalent (M/F=48%; M=44%; F=55%), and muscle pain (myalgia) being the most prevalent type (M/F=38%; M=39%; F=35%; p>0.05). M presented significant differences about M regarding the site of the injury (foot: M=6%; F=25%; p=0.035) and type of injury (intense nonspecific pain: M=28%; F=5%; p=0.040).

**Conclusion:**

Elite footvolley athletes in Brazil presented a high prevalence of injuries, with the most common injuries being in the knees. *Level of Evidence IV; Case Series.*

## RESUMO

**Objetivo:**

O futevôlei é um esporte que vem ganhando popularidade, porém a literatura sobre lesões nessa modalidade é bastante escassa. Assim, o objetivo deste estudo foi descrever as localizações e os tipos de lesões mais comuns em atletas profissionais de futevôlei e comparar a prevalência entre homens (H) e mulheres (M).

**Métodos:**

Estudo observacional, transversal, incluindo atletas profissionais mais bem classificados no Brasil no ano de 2023. As entrevistas ocorreram durante as etapas do campeonato nacional profissional de futevôlei e incluíram perguntas sobre características da amostra, histórico e rotina de treinamento, e lesões sofridas ao longo da carreira.

**Resultados:**

Participaram 56 atletas, sendo 36 M (26±5 anos; 9±4 anos de prática) e 20 M (28±6 anos; 7±3 anos de treinamento). Desse total, 95% relataram alguma lesão causada pela prática do futevôlei, sendo as lesões no joelho as mais prevalentes (H/M=48%; H=44%; M=55%; p>0,05) e a dor muscular (mialgia) o tipo mais prevalente (H/M=38%; H=39%; M=35%; p>0,05). Diferenças significativas foram verificadas nas comparações entre H e M quanto ao local da lesão (pé: H=6%; M=25%; p=0,035) e ao tipo de lesão (dor intensa inespecífica: H=28%; M=5%; p=0,040).

**Conclusão:**

Atletas de futevôlei de elite no Brasil apresentaram alta prevalência de lesões, sendo as lesões mais comuns nos joelhos. *Nível de Evidência IV; Série de Casos.*

## INTRODUCTION

Footvolley is a sport that originated on the beaches of Rio de Janeiro, Brazil, during the military dictatorship in the 1960s. As a result of the prohibition on engaging in sports that lacked a defined area and did not involve the use of a net, football players started utilizing volleyball spaces for their practice sessions.^1^ Footvolley gained popularity in the subsequent years, and its popularity was further increased during the COVID-19 pandemic. This was primarily because footvolley involves minimal physical contact between players, has a limited number of participants, and is played outdoors. These factors made footvolley a sport with a safety protocol during the pandemic.^2^

As footvolley has grown in popularity, specialized spaces dedicated to the sport have emerged. Various urban centers in Brazil have started to establish spaces for footvolley.^3^ This dissemination has contributed to the advancement of footvolley movements and techniques.^4^ However, it has also led to an increase in injuries, as both amateur practitioners and professional athletes exert oneself to keep up with the sport's evolution and attempt more complex moves, including jumps, spins, and new attacks.

Although footvolley has gained popularity and seen advancements, there is a lack of scientific research on the occurrence of injuries related to its practice.^5^ The International Olympic Committee, which is the primary regulative institution for sports, prioritizes the protection of athletes’ health.^6^ In addition, the World Health Organization acknowledges the health advantages of populations that engage in regular physical activity.^7^ Therefore, conducting research on the frequency of sports-related injuries is an essential step in suggesting preventive strategies for sports injuries,^8^ which can be advantageous for elite athletes as well as casual and non-professional footvolley players.

Notably, other sports played on sand spaces, known as beach sports, have provided data on the frequency of injuries, as demonstrated in a study conducted during the Asian Beach Games, which involved athletes from over 40 countries. According to Al-shaqsi et al,^8^ a significant number of injuries were found among professional athletes in beach handball (21%), beach soccer (15%), and beach volleyball (13%). As far as we know, there is only one study on footvolley that was conducted in 2015.^5^ This study found that 39% of the participants evaluated had footvolley injuries, with a higher occurrence of injuries in men.

Considering what has been described, our study aimed to assess the prevalence of injuries in professional footvolley athletes in Brazil, taking into account the lack of available information, and the significance of obtaining descriptive data as a crucial initial step in developing effective injury prevention strategies.^9^ Furthermore, we aimed to assess the incidence of injuries in both males and females and elucidate the primary anatomical site where these injuries commonly occurred.

## MATERIAL AND METHOD

### Study design

This study was conducted in São Paulo, Brazil, using a retrospective observational cross-sectional design. The study period spanned from August of 2023 to August of 2024. This study was approved my Local ethical committee (protocol number 6.131.376)

### Sample

We reached out to athletes who were participating in professional footvolley championships in São Paulo city (Brazil), both in person before their matches and online through WhatsApp and Instagram. Following their initial interest in participating, the athletes were subsequently approached to complete the questionnaire administered by Google Forms during national competitions.

The selection of these athletes refers to the professional ranking of the Open Nacional de Footvolley championship and the ranking of the Brazilian Footvolley League, according to the position of the athletes until the day of data collection. In short, the sample consists of athletes ranked among the top 50 in each of the aforementioned rankings. We highlight that at the time of data collection for this research, the Brazilian Footvolley Confederation had championships suspended, as in the last election there was a legal challenge, which justifies the choice of these rankings mentioned above.

### Survey of injury

Retrospective injury assessment was performed based on the International Olympic Committee guidelines for recording and reporting epidemiological data on injuries.^10^ In summary, a questionnaire with 22 questions, with questions about the characteristics of the sample (name, age, weight, height, length of time in the sport in years), aspects of training and competitions (Training volume, number of training sessions per week, being divided between footvolley and specific training to improve performance other than on the sand, average number of championships per month and carrying out mobility and/or strengthening before entering the court in championships) and occurrence of injuries (injuries that the athlete suffered during practicing the sport that took the athlete away from the sport for at least one day, with options for body parts to select and the most serious types of injury, that is, the one that kept the athlete away from the sport for the longest time, once again considering the location and type of injury; moment of injury, time away from footvolley after diagnosis and reason); Finally, questions about the athlete's perception of the greatest influence on the occurrence of the injury.

### Statistical analysis

The data is displayed in absolute frequency (n) and relative frequency (%), as well as the mean ± standard deviation (SD). The data's normality and equality of variance were evaluated using the Shapiro-Wilk and Levene tests, respectively. The study participants were categorized into two groups according to their biological sex, specifically males and females. A chi-square test was employed to compare groups (male and female) for each category variable. The Mann-Whitney test was employed to compare groups when dealing with continuous variables.

## RESULTS

The investigation comprised a total of 56 athletes. Table 1 displays the sample characteristics. The study had 36 male and 20 female participants. The athletes in question had an average age of 27.2 ± 5.6 years, a body weight of 70.9 ± 10.0 kg, and a BMI of 23.3 ± 2.0 kg/m^2^. Regarding the practice of the modality, the average duration was 8.5 ± 3.9 years, with training sessions conducted 4.0 ± 1.2 days per week. Approximately 95% of the participants in the sample reported having had injuries while playing footvolley. The duration of absence from playing footvolley due to the most severe injury was 100 ± 81 days. Upon analyzing the data from the table, it is evident in the last column that there is a statistically significant difference (p<0.05) in weight, height, BMI, and number of championships played. These values are lower for females in comparison to males.

**Table 1:** Sample characteristics.

Variable	All (n=56)		Men (n=36)	Women (n=20)	P (CI 95%)
	Mean ± DP	Min - Max	Mean ± DP	Mean ± DP	
Age (years)	27.2 ± 5.6	18 - 44	26.6 ± 5.4	28.4 ± 6.0	n.s
Weight (kg)	70.9 ± 10.0	50 - 95	76.1 ± 7.1	61.4 ± 6.9*	<0.001 (10.7 - 18.6)
Height (cm)	174 ± 9	150 - 193	180 ± 1	160 ± 6*	<0.001 (0.11 - 0.18)
BMI	23.3 ± 2.0	19 - 28	23.7 ± 1.8	22.6 ± 2.1*	0.033 (0.1 - 2.3)
Practice time (years)	8.5 ± 3.9	4 - 15	9.0 ± 4.1	7.7 ± 3.3	n.s
Days you train on court per week (days)	4.0 ± 1.2	3 - 7	4.1 ± 1.2	3.7 ± 1.3	n.s
On-court training periods per week	5.6 ± 2.9	4 - 12	6.0 ± 2.7	4.9 ± 3.2	n.s
Days you train off the court per week (days)	3.4 ± 1.5	0 - 7	3.2 ± 1.5	3.8 ± 1.4	n.s
Periods you train outside on the court per week	2.3 ± 1.1	0 - 7	1.5 ± 0.6	1.3 ± 0.6	n.s
Professional championships per month	2.4 ± 1.0	1 - 4	2.8 ± 0.8	1.6 ± 0.9	<0.001 (0.7 - 1.7)
I've already had an injury from playing footvolley	95%	--	95%	95%	--
Time away from the worst injury you had (days)	100 ± 81	2 - 210	94.3 ± 78.8	111 ± 87	n.s

Legend: SD = standard deviation; Min= minimum; Max = maximum; BMI = body mass index. CI = Confidence interval.

Table 2 displays the locations where athletes reported experiencing injuries while participating in footvolley. Upon analyzing the overall outcomes, it was found that out of the 17 distinct areas, 14 were identified as sites of injuries. The knee was the most prevalent location, with 27 athletes (48%) reporting injuries in that area. This was followed by the hip, with 18 athletes (32%) experiencing injuries, and the lumbar region, with 17 athletes (30%) reporting injuries. When examining injuries in men, the knee is the most frequently affected area, with 16 athletes (44%) experiencing injuries in this region. The lumbar region is the second most common area of injury, with 12 athletes (33%) affected. Within the female population, the knee is the most prevalent outcome, observed in 11 athletes (55%), closely followed by the hip, which is observed in 8 athletes (40%).

**Table 2:** Location of injuries suffered throughout their lives playing footvolley.

Region	All (n=56)	Men (n=36)	Women (n=20)	Qui-square
Shoulder – n (%)	10 (18%)	7 (19%)	3 (15%)	n.s.
Arm – n (%)	1 (2%)	1 (3%)	0 (0%)	n.s.
Forearm – n (%)	0 (0%)	0 (0%)	0 (0%)	--
Elbow – n (%)	4 (7%)	2 (6%)	2 (10%)	n.s.
Handle – n (%)	2 (4%)	1 (3%)	1 (5%)	n.s.
Hand – n (%)	0 (0%)	0 (0%)	0 (0%)	--
Chest – n (%)	0 (0%)	0 (0%)	0 (0%)	--
Abdomen – n (%)	1 (1%)	1 (3%)	0 (0%)	n.s.
Lumbar region – n (%)	17 (30%)	12 (33%)	5 (25%)	n.s.
Cervical region – n (%)	13 (23%)	9 (25%)	4 (20%)	n.s.
Hip – n (%)	18 (32%)	10 (28%)	8 (40%)	n.s.
Thigh (anterior) – n (%)	11 (20%)	8 (22%)	3 (15%)	n.s.
Posterior thigh – n (%)	12 (21%)	9 (25%)	3 (15%)	n.s.
Knee – n (%)	27 (48%)	16 (44%)	11 (55%)	n.s.
Calf – n (%)	8 (14%)	4 (11%)	4 (20%)	n.s.
Ankle – n (%)	14 (25%)	8 (22%)	6 (30%)	n.s.
Foot – n (%)	7 (13%)	2 (6%)	5 (25%)	0.035

Legends: n = number of athlets; % = percentual of athlets.

In Table 3 we present the types of injuries. In relation to all athletes, we can observe that muscle pain (myalgia) is the most prevalent injury (21 athletes – 38%), followed by sprain (20 athletes – 36%). When considering male injuries, muscle pain (myalgia) was the most prevalent (14 athletes - 39%) followed by sprain (11 athletes - 31%). In female professional athletes, the highest prevalence was sprain (9 athletes – 45%), followed by tendonitis (7 athletes – 35%) or muscle pain (myalgia) (7 athletes – 35%). In comparing the number of injuries by type of injuries, male vs female presented statistical differences (p=0.035) on site foot.

**Table 3:** Type of injuries suffered throughout their lives playing footvolley.

Type	All (n=52)	Men (n=34)	Women (n=20)	Qui-square
Muscle tear (muscle strain) – n (%)	13 (23%)	7 (19%)	6 (30%)	n.s.
Ligament/meniscus tear – n (%)	12 (21%)	7 (19%)	5 (25%)	n.s.
Sprain – n (%)	20 (36%)	11 31%)	9 (45%)	n.s
Tendinitis – n (%)	16 (29%)	9 (25%)	7 (35%)	n.s.
Synovitis – n (%)	0 (0%)	0 (0%)	0 (0%)	--
Bursitis – n (%)	3 (5%)	1 (3%)	2 (10%)	n.s
Periostitis – n (%)	10 (18%)	6 (17%)	4 (20%)	n.s
Fracture – n (%)	1 (2%)	0 (0%)	1 (5%)	n.s.
Muscle pain (myalgia) – n (%)	21 (38%)	14 (39%)	7 (35%)	n.s.
Severe non-specific pain – n (%)	11 (20%)	10 (28%)	1 (5%)	0.040
Nonspecific chronic pain – n (%)	10 (18%)	5 (14%)	5 (25%)	n.s.

Legends: n = number of athlets; % = percentual of athlets.

In Table 4 we present data on the most serious injury that athletes have suffered throughout their lives playing footvolley, considered by the greatest distance from the courts or those that bothered them until data collection. A total of 52 athletes who reported an injury while practicing footvolley, 48 reported a serious injury, 32 male and 16 female. The comparison of number of injuries by the site of injury, considering male vs female showed no statistical differences (p>0.05).

**Table 4:** Location of the most serious injury ever suffered while playing footvolley.

Region	All (n=48)	Men (n=32)	Women (n=16)	Qui-square
Shoulder – n (%)	2 (4%)	2 (6%)	0 (0%)	--
Arm – n (%)	0 (0%)	0 (0%)	0 (0%)	--
Forearm – n (%)	0 (0%)	0 (0%)	0 (0%)	--
Elbow – n (%)	0 (0%)	0 (0%)	0 (0%)	--
Handle – n (%)	1 (2%)	1 (3%)	0 (0%)	--
Hand – n (%)	0 (0%)	0 (0%)	0 (0%)	--
Chest – n (%)	0 (0%)	0 (0%)	0 (0%)	--
Abdomen – n (%)	0 (0%)	0 (0%)	0 (0%)	--
Lumbar region – n (%)	3 (6%)	2 (6%)	1 (6%)	n.s.
Cervical region – n (%)	1 (2%)	1 (3%)	0 (0%)	--
Hip – n (%)	10 (21%)	6 (19%)	4 (25%)	n.s.
Thigh (anterior) – n (%)	5 (10%)	4 (12%)	1 (6%)	n.s.
Posterior thigh – n (%)	2 (4%)	2 (6%)	0 (0%)	--
Knee – n (%)	16 (33%)	9 (28%)	7 (44%)	n.s.
Calf – n (%)	0 (0%)	0 (0%)	0 (0%)	--
Ankle – n (%)	6 (13%)	4 (13%)	2 (13%)	n.s.
Foot – n (%)	2 (4%)	1 (3%)	1 (6%)	n.s.

Legends: n = number of athlets; % = percentual of athlets.

As reported for the most common injuries (Table 2), the knee is the most serious site of injury for both sexes (16 athletes – 33%), followed by the hip (10 athletes – 21%). For men (n=9; 28%) the knee was the site of the most serious injury, as well as for female (n=7; 44%). Finally, male athletes presented the hip region as the second region with the most injuries (06 athletes – 19%), as did female (4 athletes – 25%). The comparison of the location of the most serious injury ever suffered while playing footvolley, considering male vs female showed no statistical differences (p>0.05).

Finally, in Table 5 we present the most serious types of injuries. Injuries due to ligament/meniscus tears were the most reported (total: n=10, 21%; men: n= 6, 19%; female: n=4, 25%), followed by muscle tear, muscle strain (total: n=7, 15%; men: n=6, 19%; women: n=1, 6.3%). In comparing the number of type of most serious injuries suffered throughout their lives playing footvolley, considering male vs female was verified no statistical differences (p>0.05).

**Table 5:** Type of most serious injuries suffered throughout their lives playing footvolley.

Type	All (n=48)	Men (n=32)	Women (n=16)	Qui-square
Muscle tear/strain – n (%)	7 (15%)	6 (19%)	1 (6%)	n.s
Ligament/meniscus tear – n (%)	10 (21%)	6 (19%)	4 (25%)	n.s
Sprain – n (%)	3 (6%)	2 (6%)	1 (6%)	n.s
Tendinitis – n (%)	4 (8%)	2 (6%)	2 (12%)	n.s
Synovitis – n (%)	0 (0%)	0 (0%)	0 (0%)	--
Bursitis – n (%)	0 (0%)	0 (0%)	0 (0%)	--
Periostitis – n (%)	0 (0%)	0 (0%)	0 (0%)	--
Fracture – n (%)	1 (2%)	0 (0%)	1 (6%)	n.s
Muscle pain (myalgia) – n (%)	3 (6%)	2 (6%)	1 (6%)	n.s
Severe non-specific pain – n (%)	6 (13%)	5 (16%)	1 (6%)	n.s
Nonspecific chronic pain – n (%)	7 (15%)	3 (9%)	4 (25%)	n.s

Legends: n = number of athlets; % = percentual of athlets.

## DISCUSSION

The present study aimed to assess the prevalence of injuries in both male and female professional footvolley athletes in Brazil, and elucidate the primary anatomical site where these injuries commonly occurred. A total of 14 different areas were injured, with the knee being the most common site with a prevalence of 48%, followed by the hip with a prevalence of 32%, and the lumbar region with a prevalence of 30%. In a comparison of males and females, we identified differences in the location of injuries suffered throughout their lives playing footvolley (foot – male 6% vs female 25% p=0.035) and type of injuries suffered throughout their lives playing footvolley (Severe non-specific pain – male 28% vs female 5% p=0.04).

In comparison to the 2015 research on footvolley athletes, the percentage of total athletes who reported previous injuries was 41% and now 86%, indicating a variation in the occurrence of injuries in footvolley. In addition, even though the Asian Beach Games study did not include the footvolley modality, our observed prevalence of injuries was higher than seen in beach volleyball players (13%) and beach soccer athletes (24%).^8^

When comparing our data with a study conducted by De Menezes et al.^11^ on the most common injuries in men's beach volleyball, we found that the knee and lumbar region were the areas with the highest number of injuries. Regarding the Asian Beach Games research, the knee and lumbar region were the most often injured anatomical locations in beach volleyball athletes, accounting for 21% of injuries each. In contrast, beach soccer had the foot as the primary source of injury, accounting for 37% of injuries.^8^ According to research conducted by Alves and colleagues, the lumbar region (56%) and the spine (32%) are the most afflicted locations in footvolley. Specifically, the knee has a frequency of 26% and the lumbar region has a prevalence of 24%.^5^

When comparing injuries between male and females, a significant disparity in answers is observed regarding non-specific severe pain. Specifically, 28% of male athletes presented non-specific severe pain, but just 5% of female athletes did so. This outcome can be somewhat elucidated by the observation that women possess a superior capacity to differentiate pain feelings in comparison to males.^12^ The study conducted during the Asian Beach Games revealed that sprain is the predominant injury among beach volleyball participants, accounting for 50% of the cases. In beach soccer, strain was the most prevalent injury, accounting for 21% of the cases, followed by sprain at 19%.^8^

It is crucial to emphasize that, concerning the most severe injuries, we inquired the athletes about the precise moment when these injuries happened and obtained the following outcomes: 41% of the participants were engaged in a tournament, while 22% were involved in footvolley training. Additionally, 23% were actively playing footvolley, and 14% were doing specialized training sessions outside of the sand. A study conducted on professional football athletes from the eight main teams that participated in the Campeonato Paulista Series A2 in 2010 revealed a significant incidence of injuries during the championship. Approximately 61% of the players experienced at least one injury during this period.^13^

The athlete's most severe injuries were found to be influenced by several factors. These include physical exhaustion resulting from excessive training, competitions, or games (45% of reports), inadequate treatment of previous injuries (16%), ground conditions and climate (11%), insufficient heating (9%), and mental exhaustion (8%). The higher incidence of injuries observed during championships, coupled with the significant impact of physical fatigue, underscores the need for a comprehensive study to raise awareness among professional athletes regarding the extent of strain they face due to the increasing number of championships and, consequently, more rigorous game preparations. It is crucial to engage with expert health and sports specialists to tailor the whole preparing time for the championship, considering the specific requirements of the sport and any recent developments.^14^

This study is not free of limitations. Initially, we employed self-reported data. This type of data may have biases in responses due to the athletes’ recollections. An optimal scenario would have a cohort design that includes a comprehensive registry of all athlete injuries spanning many years. Despite this constraint, it is crucial to emphasize the challenges associated with implementing the cohort design among athletes, particularly in sports with less popularity, such as footvolley. Additionally, the small sample size are important limitations. Furthermore, the limited sample size is a significant restriction. This may have resulted in an overestimation of the impact. Subsequent investigations should validate these conclusions by the implementation of more extensive studies.

## CONCLUSION

Our study revealed that elite footvolley athletes in Brazil experienced injuries in 14 distinct anatomical regions, with the knee being the most prevalent site, followed by the hip and lower back. Upon comparing male and female athletes, we observed disparities in the occurrence of injuries sustained over their footvolley careers. Notably, female athletes exhibited a greater incidence of foot injuries compared to their male counterparts. Male athletes had a greater incidence of severe and non-specific pain in comparison to their female counterparts, in terms of the nature of the injuries sustained.
